# Combined autologous chondrocyte implantation (ACI) with supra-condylar femoral varus osteotomy, following lateral growth-plate damage in an adolescent knee: 8-year follow-up

**DOI:** 10.1186/1758-2555-3-5

**Published:** 2011-03-18

**Authors:** Sridhar Vijayan, George Bentley

**Affiliations:** 1 Joint Reconstruction and Cartilage Transplantation Unit, Royal National Orthopaedic Hospital, Brockley Hill, Stanmore, HA7 4LP, UK

## Abstract

We report the 8-year clinical and radiographic outcome of an adolescent patient with a large osteochondral defect of the lateral femoral condyle, and ipsilateral genu valgum secondary to an epiphyseal injury, managed with autologous chondrocyte implantation (ACI) and supracondylar re-alignment femoral osteotomy. Long-term clinical success was achieved using this method, illustrating the effective use of re-alignment osteotomy in correcting mal-alignment of the knee, protecting the ACI graft site and providing the optimum environment for cartilage repair and regeneration. This is the first report of the combined use of ACI and femoral osteotomy for such a case.

## Background

Injury to long bones in the lower extremities with involvement of the pyseal growth-plate is common in children and adolescents [1]. It is estimated that 15% of all long bone injuries during childhood involve the physeal growth-plate [2]. Chondral damage to the knee is common and retrospective reviews of large numbers of arthroscopic procedures have shown a prevalence of between 63% to 67% of focal chondral or osteochondral lesions [3,4]. Breakdown of articular cartilage secondary to trauma or disease results in severe pain and disability, ultimately progressing to early onset osteoarthritis [5,6]. The use of corrective high tibial osteotomy [7-9] and chondrocyte transplantation [10-12] for the treatment of isolated unicompartmental osteochondral defects of the knee are both well-described separately, as are the benefits of restoration of the mechanical axis of the knee prior to or in conjunction with chondrocyte transplantation to protect the graft site in a fully mobile knee [7,13]. The use of combined techniques for cartilage repair is becoming commoner, especially in younger patients [**Parratt MTR et al**: Chondrocyte transplantation combined with high tibial osteotomy in the treatment of osteochondral defects in the adolescent knee. 2011 Submitted].

In this case distal femoral supracondylar osteotomy, to correct a valgus deformity at the knee caused by presumed trauma-related physeal arrest and subsequent dysplasia of the lateral femoral condyle was performed following ACI for a large 4 cm × 5 cm osteochondral defect.

### Case Presentation

A 15 year-old male presented to his local Orthopaedic outpatient department complaining of a swollen painful right knee. A keen rugby player, he had also sustained an injury three months previously whilst skiing, and at the time of presentation complained of persistent pain, which had prevented him engaging in any further sporting activity. Plain radiographs confirmed the diagnosis of a stable osteochondral defect in the lateral femoral condyle. Eight months later, the patient was referred to our unit with continued pain in the right knee, unable to participate in sports. The patient, a semi-professional rugby player, was keen to return to high-level competitive sports again.

Clinical examination revealed a unilateral valgus deformity of the right knee, with a moderate effusion. He had a non-reciprocating stair-climbing gait. Range of movement was 0 to 140°. There was marked wasting of the right quadriceps. Patellar tracking and ligament examination was normal.

**Figure 1:**
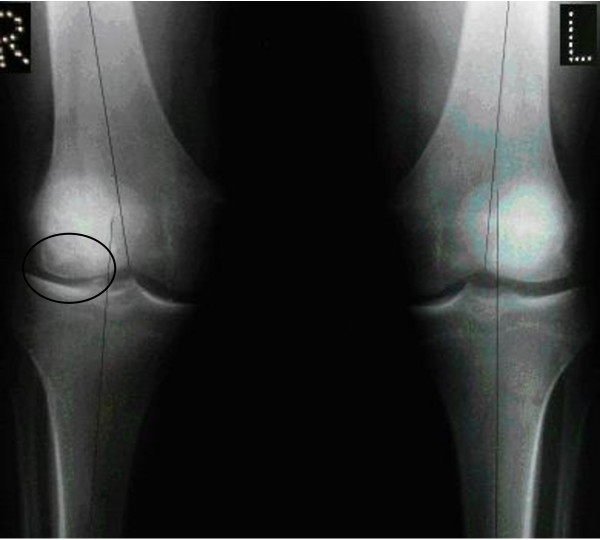
**Pre-operative antero-posterior weight-bearing radiograph of both knees showing a lateral femoral condyle articular cartilage lesion (circled) and a valgus deformity of 11 degrees on the right side with hypoplasia of the lateral femoral condyle, compared with 5 degrees on the left**.

Standing AP radiographs showed a valgus alignment of 11 degrees on the right and 5 degrees on the left and an abnormality of the right lateral femoral condyle (Figure 1). Magnetic resonance imaging confirmed a full-thickness osteochondral lesion on the lateral femoral condyle measuring approximately 5 cm^2^. All of this was in keeping with a diagnosis of Osteochondritis dissecans.

His metabolic and haematological parameters were within the normal range. Pre-operatively, the patient's function was assessed using the Modified Cincinnati score [14] (40 out of 100) and Bentley functional rating system [15] (4 out of 5). Pain assessed using the visual analogue score (VAS) was 9 out of 10.

**Figure 2:**
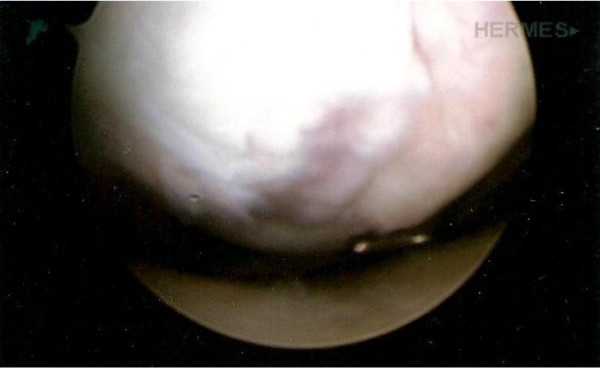
**Arthroscopic appearance of lateral femoral condyle showing surface cartilage fragmentation and irregularity of the subchondral bone**.

Arthroscopic assessment showed an extensive articular osteochondral defect with gross irregularity of the articular surface of the lateral femoral condyle measuring 4 × 5 cm (Figure 2), equivalent to a grade IV on the International Cartilage Repair Society (ICRS) scale. In view of the extent of these findings it was decided to attempt to repair the defect by autologous chondrocyte implantation (ACI). Because the valgus deformity was not very severe and the knee had a range of movement of only 10-90°, the ACI was performed first. Full thickness cartilage was harvested from the margin of the lateral femoral trochlea and sent for chondrocyte culture. At second stage surgery five weeks later, a total of 8 million expanded cultured chondrocytes were implanted beneath a porcine derived type I/III collagen membrane covering the large lateral femoral condyle defect via a medial arthrotomy. Post-operatively the patient was allowed to weight-bear in a cylinder cast for 2 weeks and underwent intensive protected low-impact physiotherapy.

After six months of protected weight-bearing and active mobilisation, when he had recovered from the chondrocyte transplantation procedure, the valgus deformity was corrected to restore the mechanical axis. This was achieved by a supracondylar medial closing-wedge femoral osteotomy and internal fixation with a blade-plate. At surgery it was aimed to correct the deformity to a physiological alignment of +3 degrees with the intention of off-loading the ACI graft. The metalwork was removed twelve months later and the patient was permitted to engage in competitive sports including rugby and football.

Modified Cincinnati, Bentley and VAS scores were taken at 12 and 24 months post-operatively. Modified Cincinnati scores were 60 and 76, and Bentley score was 3 and 2. VAS scores were 6 and 3 respectively.

Subsequent clinical assessment at eight years post-operatively, the patient complained of no pain, locking or giving way of the knee; he is able to kneel and squat without pain and has been able to resume full sporting activities including semi-professional rugby at university, skiing and free-weight gym activity. The right knee had a full range of unrestricted movement, there was no evidence of quadriceps wasting and there was equal leg length. His Modified Cincinnati Score was 92, Bentley score 0 and VAS 0.

**Figure 3:**
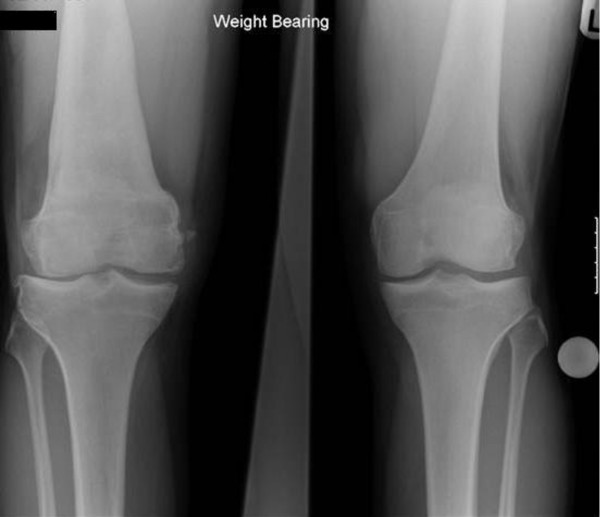
**Antero-posterior weight-bearing radiograph of both knees 8 years after ACI right lateral femoral condyle and supracondylar varus osteotomy showing neutral alignment and joint space narrowing laterally**.

**Figure 4:**
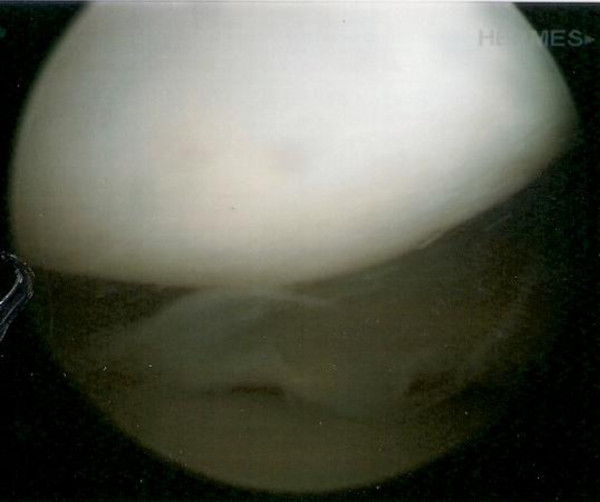
**Arthroscopic appearance of lateral femoral condyle 8 years post-operatively showing a well-healed osteochondral defect from ACI**.

Subsequently in the year, the patient presented with right knee pain secondary to a recent sports injury. Latest radiographic assessment revealed a neutral alignment of the right leg, with joint space narrowing laterally, new-spur formation and a well-healed osteochondral lesion (Figure 3). Continued pain warranted an arthroscopy, which showed a minor tear in the lateral meniscus. The lateral femoral condyle demonstrated an intact ACI graft with good cartilage growth (Figure 4). After debridement and trimming of the lateral meniscus the patient was pain free.

## Discussion

Physeal injuries in the lower extremity are most common in the proximal tibia and the distal femur affecting the knee [16,17]. Such injuries can result in deformities of the lower limb, leading to secondary compartmental osteoarthritis in adjacent joints due to deviation of the mechanical axis [18,19]. Biomechanical malalignment may lead to increased compressive loading of the joint, thereby damaging induced repair tissue produced by cartilage regeneration techniques such as ACI [13].

The successful use of ACI for the treatment of large isolated osteochondral defects of the femoral condyle, patella and trochlea is well-reported in medium-term review [20-23]. Distal femoral osteotomy for the correction and realignment of valgus deformity [8,24,25] as well as for the treatment of lateral-compartment osteoarthritis of the knee [25] has also been well described. Generally it is considered that correction of mal-alignment is preferable before proceeding with chondrocyte implantation to avoid excessive loading on the graft site [26,27]. Successful correction of tibial mal-alignment is most commonly performed by high tibial osteotomy prior to or in conjunction with chondrocyte transplantion, and has been shown to produce significant improvement in subjective assessment in medium-term review [[8], **Parratt MTR et al**: Chondrocyte transplantation combined with high tibial osteotomy in the treatment of osteochondral defects in the adolescent knee. 2011 Submitted].

Management of severe deformities resulting from physeal arrest with re-alignment osteotomy are also well described for example by the use of tibial valgus osteotomy for tibia vara in Blount's disease [28]. However genu valgum following femoral physeal injury is rare [29].

In this case we demonstrated the development of moderate valgus deformity as a consequence of lateral physeal arrest and resultant imbalance in condylar development, in a patient who became symptomatic from a co-existing osteochondral femoral condylar defect. It is possible that a single traumatic incident was responsible for both pathologies. The history of very active sport during the growth period suggests that the injury occurred some years before presentation.

Considering the patient's symptoms, the relatively mild deformity, and the restricted range of movement, it was considered that the most appropriate management was firstly to treat the symptomatic osteochondral defect and mobilize the knee. Subsequent restoration of the mechanical alignment by a varus osteotomy at the level of the deformity in the distal femur using the medial closing-wedge technique followed. Currently, no evidence exists that such a large defect could heal spontaneously with osteotomy alone, yet it cannot be concluded that ACI alone was responsible for the patient's symptomatic improvement. Therefore, in view of his youth, everything possible to produce healing of the extensive cartilage defect was thought necessary, hence the combined use of ACI with staged osteotomy.

## Competing interests

The authors declare that they have no competing interests.

## Authors' contributions

All authors co-wrote the paper and discussed the results for the manuscript preparation. All authors have read and approved the final manuscript.

## Consent

Verbal informed consent was obtained from the patient for publication of this case report and the images.
